# Contact and Repellent Activities of the Essential Oil from *Juniperus formosana* against Two Stored Product Insects

**DOI:** 10.3390/molecules21040504

**Published:** 2016-04-16

**Authors:** Shanshan Guo, Wenjuan Zhang, Junyu Liang, Chunxue You, Zhufeng Geng, Chengfang Wang, Shushan Du

**Affiliations:** 1 Beijing Key Laboratory of Traditional Chinese Medicine Protection and Utilization, Beijing Normal University, Beijing 100875, China; guoshanshan@mail.bnu.edu.cn (S.G.); zwj0729@mail.bnu.edu.cn (W.Z.); liangjunyu@nwnu.edu.cn (J.L.); youchunxue@mail.bnu.edu.cn (C.Y.); gengzhufeng@bnu.edu.cn (Z.G.); wangchengfang@mail.bnu.edu.cn (C.W.); 2 Analytical and Testing Center, Beijing Normal University, Beijing 100875, China; 3 China CDC Key Laboratory of Radiological Protection and Nuclear Emergency, National Institute for Radiological Protection, Chinese Center for Disease Control and Prevention, Xicheng District, Beijing 100088, China

**Keywords:** *Juniperus formosana*, *Tribolium castaneum*, *Liposcelis bostrychophila*, contact toxicity, repellency

## Abstract

The chemical composition of the essential oil from *Juniperus formosana* leaves and its contact and repellent activities against *Tribolium castaneum* and *Liposcelis* *bostrychophila* adults were investigated. The essential oil of *J. formosana* leaves was obtained by hydrodistillation and analyzed by GC-MS. A total of 28 components were identified and the main compounds in the essential oil were α-pinene (21.66%), 4-terpineol (11.25%), limonene (11.00%) and β-phellandrene (6.63%). The constituents α-pinene, 4-terpineol and d-limonene were isolated from the essential oil. It was found that the essential oil exhibited contact activity against *T. castaneum* and *L. bostrychophila* adults (LD_50_ = 29.14 μg/adult and 81.50 µg/cm^2^, respectively). The compound 4-terpineol exhibited the strongest contact activity (LD_50_ = 7.65 μg/adult). In addition, data showed that at 78.63 nL/cm^2^, the essential oil and the three isolated compounds strongly repelled *T. castaneum* adults. The compounds α-pinene and d-limonene reached the same level (Class V) of repellency as DEET (*p* = 0.396 and 0.664) against *L. bostrychophila* at 63.17 nL/cm^2^ after 2 h treatment. The results indicate that the essential oil and the isolated compounds have potential to be developed into natural insecticides and repellents to control insects in stored products.

## 1. Introduction

*Juniperus formosana* Hayata (Pinales: Cupressaceae), an evergreen shrub or tree, is endemic to China and widely distributed in nearly 14 provinces [1]. *J. formosana* is generally considered as a medicinal plant. Its roots, leaves and branches are used in traditional Chinese medicine to cure measles, scabs and fever. Furthermore, the fruits and fresh branches with leaves from *J. formosana* are used in traditional Tibetan medicine for treatment of pruritus cutanea, hemorrhoids, deep-rooted boils and anthrax [2,3].

Currently, the use of synthetic chemicals to control insects and arthropods raises several concerns related to the environment and human health. An alternative is to use natural products that possess good efficacy and are environmentally friendly [4,5]. Among them, the essential oils derived from many medicinal aromatic plants have received much attention and have been thoroughly investigated as natural insecticides against several stored product insects [6,7,8,9]. As a consequence, this vast arsenal of bioactive compounds has attracted significant and increasing attention of researchers in recent years [10]. During our screening program for new agrochemicals from Chinese medicinal herbs [11,12,13,14,15], the essential oil from *J. formosana* was found to possess contact and repellent activities towards the red flour beetle, *Tribolium castaneum* Herbst (Coleoptera: Tenebrionidae), and the booklouse, *Liposcelis bostrychophila* Badonnel (Psocoptera: Liposcelididae). The red flour beetle, found wherever grains or other dried foods are stored, has a highly evolved kidney-like cryptonephridial organ to survive such extremely dry environments. It has demonstrated resistance to all classes of insecticides used against it [16]. The booklouse is found in stored-product grains and traditional medicines, or other amylaceous products [17]. It is a tiny (approximately 1 mm in length), wingless, light brown insect, and is regarded as a secondary pest, often overlooked due to its small size and the existence of other, more damaging primary pests (e.g., maize weevils *Sitophilus zeamais*, rice weevils *Sitophilus oryzae* and the lesser grain borer *Rhyzopertha dominica*) in cereal grains [18].

The essential oils of other plants from the *Juniperus* genus have been demonstrated to possess bioactivity against several insects, e.g., *Anopheles stephensi*, *Aedes aegypti*, *Culex quinquefasciatus* [19], *S. oryzae* [20,21], *T. castaneum* [21,22], *Pseudaletia unipuncta* [23], *Xenopsylla cheopis* [24], *Resseliella oculiperda* [25], *Reticulitermes speratus* [26] and *Acanthoscelides obtectus* [27]. However, there is no reporting on the insecticidal or repellent activity of the essential oil from *J. formosana* against *T. castaneum* and *L. bostrychophila*. Thus, this work is undertaken to investigate the contact and repellent activities of the essential oil against the two stored product insects for the first time.

## 2. Results and Discussion

### 2.1. Essential Oil Chemical Composition

The *J. formosana* essential oil was light green with a yield of 0.46% (*v*/*w*) and density of 0.94 g/mL. The retention index (RI), compounds, percentage and identification methods were indicated in Table 1. The compounds were listed on the basis of their RI. The main constituents of the essential oil were α-pinene (21.66%) and 4-terpineol (11.25%), followed by limonene (11.00%) and β-phellandrene (6.63%). A total of 28 components were identified, accounting for 83.93% of the total oil.

Some literature is available on the chemical composition of the oils extracted from this species. The essential oil of *J. formosana* leaves obtained from the province of Gansu varied quantitatively in the amount of a-pinene (47.7%), myrcene (11.2%) and limonene (4.0%) [28]. However, although both taken from Gansu Province and tested in the same condition, the main constituents in the volatile oil obtained from Yuzhong were α-pinene (44.92%), β-caryophyllene (9.23%), isoledene (6.50%), α-humulene (5.60%), myrcene (4.54%) and d-cadinene (3.37%), while those from Lugu were di-epi-α-cedrene (31.87%), cyclohexene (15.28%), γ-elemene (10.05%), lanceol (5.80%) and α-pinene (5.79%) [29]. We hypothesized that these differences may be due to the location of the collection (sun, shadow, geographic location) and the plant physiological status. Moreover, the compositional variations can be observed in oils from different organs of the same species. The major compounds of the essential oil in the fruits of *J. formosana* grown in Kunming (Yunnan Province, China) differed qualitatively and quantitatively from our species with higher amounts of myrcene (27.08%), α-pinene (26.13%), and γ-terpinene (10.66%), but lower amounts of limonene (5.97%) and 4-terpineol (0.07%) [30], while the essential oil of *J. formosana* leaves obtained from Kunming were dominant in α-pinene (9.56%), bornyl acetate (5.17%), limonene (4.26%), myrcene (4.12%) and β-cubebene (3.88%) [2]. The essential oils from *J. formosana* grown in Taiwan were also exhibited differences between the leaf parts and fruit parts. The leaf oil mainly consisted of α-pinene (41.0%), limonene (11.5%), α-cadinol (11.0%), elemol (6.3%), and β-myrcene (5.8%), while the fruit oil was mostly α-pinene (40.9%), β-myrcene (32.4%), α-thujene (5.9%) and limonene (5.9%) [31]. In addition, the method of essential oil extraction also becomes an important factor. Extracted by supercritical-CO_2_ fluid, the essential oils from *J. formosana* contained 29 components, and 21 of them were identified and were first obtained from the plant [32]. The above findings suggest that further studies on plant cultivation and essential oil extraction standardization are needed.

**Figure 1:**
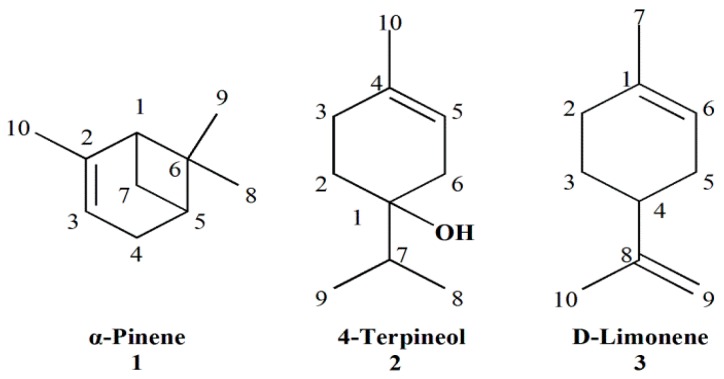
Compounds isolated from the essential oil of *J. formosana* leaves.

The compounds α-pinene (**1**), 4-terpineol (**2**) and d-limonene (**3**) were further separated and purified by silica gel column chromatography and were characterized from their ^1^H-, ^13^C-NMR (the detailed data are shown in the Supplementary Materials) and mass spectra. After comparing the physicochemical and spectrometric data with those reported in the literature [33,34,35], the identities of the three compounds were further confirmed (Figure 1).

### 2.2. Contact and Repellent Activities of the Essential Oil

The effects of contact toxicity of *Juniperus formosana* essential oil and its constituents against the two stored product insects are shown in Table 2. The essential oil of *J. formosana* leaves exhibited moderate contact toxicity against *T. castaneum* with a LD_50_ value of 29.14 µg/adult. 4-Terpineol (LD_50_ = 7.65 µg/adult) possessed stronger contact toxicity against *T. castaneum* than α-pinene and d-limonene. As for *L. bostrychophila*, the essential oil exhibited strong toxicity (LD_50_ = 81.50 µg/cm^2^) and 4-terpineol (LD_50_ = 29.50 µg/cm^2^) also possessed the strongest toxicity against the booklice among the three compounds. Compared with pyrethrum extract, the essential oil of *J. formosana* showed weaker toxicity against the two insects.

**Figure 2:**
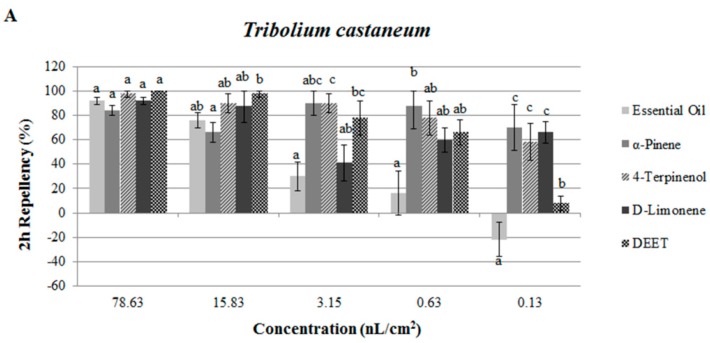

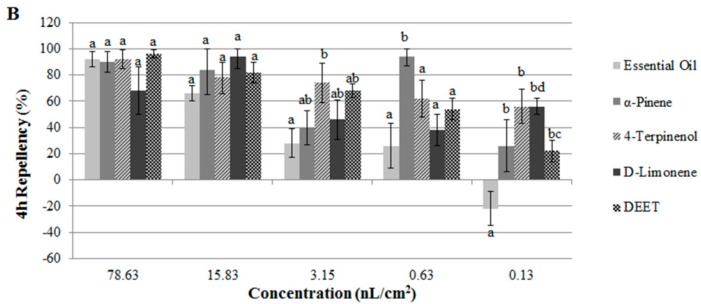
Percentage repellency (PR) of *J. formosana* essential oil and its constituents against *T. castaneum* at 2 h (**A**) and 4 h (**B**) after exposure. Means in the same column followed by the same letters do not differ significantly (*p* > 0.05) in ANOVA and Tukey’s tests. PR was subjected to an arcsine square-root transformation before ANOVA and Tukey’s tests.

**Figure 3:**
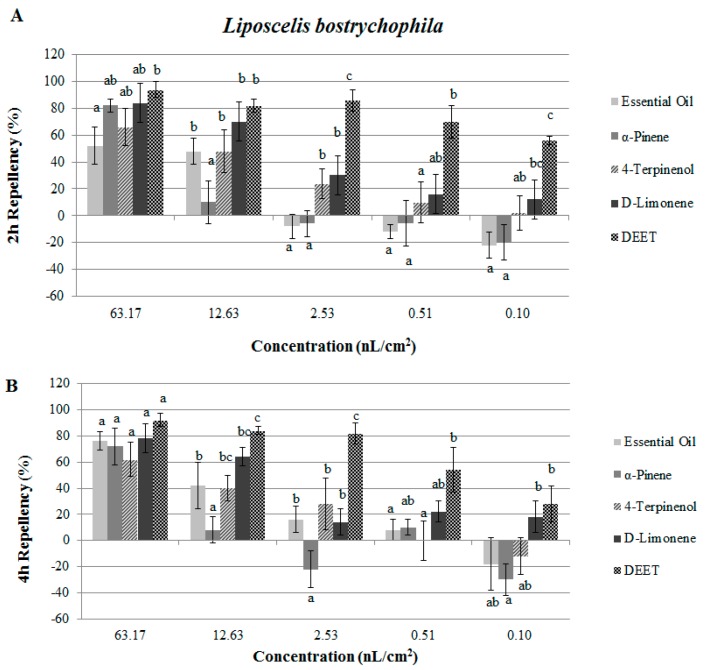
Percentage repellency (PR) of *J. formosana* essential oil and its constituents against *L. bostrychophila* at 2 h (**A**) and 4 h (**B**) after exposure. Means in the same column followed by the same letters do not differ significantly (*p* > 0.05) in ANOVA and Tukey’s tests. PR was subjected to an arcsine square-root transformation before ANOVA and Tukey’s tests.

The results of repellency assays for the essential oil and constituents against *T. castaneum* and *L. bostrychophila* are presented in Figure 2 and Figure 3. Data showed that at 78.63 nL/cm^2^, the essential oil of *J. formosana* and the three isolated compounds showed strong repellent activity against *T. castaneum* with the percentage repellency (PR) over 80% at 2 h (Figure 2). Compared with the positive control DEET, the oil, α-pinene, 4-terpineol and d-limonene showed the same level of repellency after 2 h treatment (*p* = 0.378, 0.092, 0.982 and 0.242). However, at the dose of 0.13 nL/cm^2^, the essential oil showed insect attractant properties (PR = −22%, at 2 h and 4 h), but the three isolated compounds still exhibited repellent activity against *T. castaneum*. In Figure 3, at 63.17 nL/cm^2^, the essential oil repelled *L. bostrychophila* moderately (PR = 52% and 76%, Class III and Class IV respectively, at 2 h and 4 h). When compared with the positive control, at the highest concentration, the essential oil showed moderate repellency against *L. bostrychophila*, while α-pinene and d-limonene reached the same level (Class V) of repellency with DEET (*p* = 0.396 and 0.664) against *L. bostrychophila* after 2 h treatment.

The observed results against *T. castaneum* and *L. bostrychophila* were consistent with previous reports [36,37,38]. The investigation of bioactivities of the *Litsea cubeba* and *Etlingera yunnanensis* and the individual compounds demonstrated the contact and repellent activities of α-pinene and d-limonene against *L. bostrychophila* [37,38]. Meanwhile, it has been reported that 4-terpineol has fumigant, contact and repellent activities against *T. castaneum* [39]. However, although the three isolated compounds of the tested essential oil all possessed contact and repellent activities, the bioactivities of the essential oil may be ascribed in part, but not exclusively, to the content (43.91% of the total oil) of the isolated compounds. Essential oils always represent a complex mixture of different chemical components, and thus it is very difficult to reduce the effect of the total oil to a few active principles. In this work, the essential oil showed stronger contact activity against *L. bostrychophila* than all of the three isolated compounds and the essential oil possessed a lower repellency class than they did. In addition, some compounds identified in the tested essential oil also demonstrated bioactivities against many insects. It has been reported that hedycaryol had the highest spatial and contact repellency values against *A. aegypti* [40]. Estragole was also found to have contact and repellent activities against *T. castaneum*, *L. bostrychophila* [38], *Lasioderma serricorne*, *S. oryzae*, *Callosobruchus chinensis* [41] and *S. zeamais* [42,43]. Additionally, γ-terpinene was found to exhibit activity against *T. castaneum* [44] and show high toxicity against *Anopheles sinensis*, *A. aegypti, Aedes albopictus* and *Culex pipiens pallens* mosquito larvae [45]. Moreover, germacrene D is known to have a strong effect on insect behavior [46], and α-terpineol exhibited fumigant, contact and repellent bioactivities against *L. serricorne* [47]. So the bioactivity properties of the essential oil may be related to the synergistic effects of its diverse major and minor components. Hence, isolating chemical constituents and assessing their appreciable bioactivity is important.

Because the composition of the leaf essential oils of a section of *Juniperus* contained similar simple monoterpenes [48], we hypothesized that the far-eastern junipers (*J. formosana*, *J. conferta* and *J. rigida*) may have similar insecticidal and repellent activity. According to the previous reports, based on DNA data, *J. formosana* clustered together with *J. conferta* and *J. rigida* [49]. This relationship is also seen in their essential oil compositions. Thus, further investigations also need to be conducted to evaluate the efficacy of the essential oil from the other two far-eastern junipers. Junipers are abundant resources and are widely distributed throughout the northern hemisphere [50]. This work also provides scientific bases for the exploitation and usage of junipers.

## 3. Materials and Methods 

### 3.1. Chemicals

Silica gel (200–300 mesh) and silica gel plate G were purchased from Qingdao Marine Chemical Plant (Shandong Province, China). Fluon was purchased from Beijing Sino-Rich Co. (Beijing, China). C_5_–C_36_ *n*-alkanes were purchased from Sigma-Aldrich (St. Louis, MO, USA). The positive control in contact toxicity assay, pyrethrins (pyrethrin I, 24%; pyrethrin II, 13%; cinnerin I, 2%; cinnerin II, 2%; jasmolin I, 1%; jasmolin II, 1%), were purchased from Dr. Ehrenstorfer, Germany. A commercial repellent, DEET (*N*,*N*-diethyl-3-methylbenzamide), was obtained from the National Center of Pesticide Standards (8 Shenliao West Road, Tiexi District, Shenyang, China) and used as a positive control in repellency test. All other chemicals and reagents were of analytical grade.

### 3.2. Plant Material and Essential Oil Extraction

Leaves of *J. formosana* (2 kg) were purchased from medicine market in Anguo, Hebei Province, China. The plant was identified by Dr. Liu Q.R. (College of Life Sciences, Beijing Normal University, Beijing, China) and a voucher specimen (BNU-CMH-Dushushan-2014-03-23-003) was deposited at the Herbarium (BNU) of College of Resources Science and Technology, Beijing Normal University. Then the leaves of *J. formosana* were subjected to hydrodistillation using a modified Clevenger-type apparatus for 6 h and extracted with *n*-hexane. Anhydrous sodium sulphate was used to remove water after extraction. The essential oil was stored in an airtight container in a refrigerator at 4 °C.

### 3.3. Insects

The insects were obtained from laboratory cultures maintained for the last two years in the dark incubator at 28–30 °C and 70%–80% relative humidity. *T. castaneum* were reared on wheat flour mixed with yeast (10:1, *w*/*w*) at 12%–13% moisture content and *L. bostrychophila* was reared on a 10:1:1 mixture, by mass, of flour, milk powder and yeast. Insects used in all the experiments were about one to two weeks old. All the containers housing *L. bostrychophila* and the Petri dishes used in experiments were made escape-proof with a coating of polyterafluoroethylene.

### 3.4. GC-MS and GC-FID Analyses

The volatile components of the *J. formosana* essential oil were analyzed by GC-FID and GC-MS using an Agilent 6890N gas chromatograph hooked to an Agilent 5973N mass selective detector. The same column and analysis conditions were used for both GC-FID and GC-MS. They were equipped with a HP-5MS (30 m × 0.25 mm × 0.25 μm) capillary column. The column temperature was programmed at 50 °C for 2 min, then increased to 250 °C at 2 °C/min and held for 2 min, and then increased at 10 °C/min until the final temperature of 250 °C was reached, where it was held for 5 min. The injector temperature was maintained at 250 °C and the volume injected was 1 μL of 1% solution (diluted in *n*-hexane). The helium gas used as the carrier gas at a flow rate of 1.0 mL/min. Spectra were scanned from 50 to 550 *m*/*z*. Most constituents were identified by comparison of their retention indices with those reported in the literatures. The retention indices were determined in relation to a homologous series of *n*-alkanes (C_5_–C_36_) under the same operating conditions. Further identification was made by comparison of their mass spectra with those stored in NIST 05 (Standard Reference Data, Gaithersburg, MD, USA) and Wiley 275 libraries (Wiley, New York, NY, USA) or with mass spectra from literature [51]. Relative percentages of the individual components of the essential oil were obtained by averaging the GC-FID peak area % reports.

### 3.5. Purification and Characterization of Three Compounds

The crude essential oil (7 mL) was chromatographed on a silica gel column (500 mm × 45 mm) by gradient elution with *n*-hexane first, then with *n*-hexane-ethyl acetate, and last with ethyl acetate. Fractions (200 mL) were collected and concentrated at 25 °C, and similar fractions according to thin layer chromatography (TLC) profiles were combined to yield 25 fractions. According to similar TLC profiles, fractions (5–6, 11–15) were pooled and further purified by preparative thin layer chromatography (PTLC) until obtain the pure compounds. The pure compounds were identified as α-pinene (**1**, 1.26 g), 4-terpineol (**2**, 0.84 g), d-limonene (**3**, 0.75 g). The isolated compounds were elucidated based on nuclear magnetic resonance. ^1^H- and ^13^C-NMR spectra were recorded on Bruker Avance DRX 500 instruments using CDCl_3_ as solvent with TMS as internal standard.

### 3.6. Contact Toxicity

The contact toxicity of the essential oil and the individual compounds against *T. castaneum* adults was measured as described [52]. Range-finding studies were run to determine the appropriate testing concentrations. Aliquots of 0.5 μL of the essential oil (diluted with *n*-hexane at five different concentrations) were applied topically to the dorsal thorax of the insects (10 insects per replicate, five replicates per dose). Insects treated with *n*-hexane alone were used as controls. Both treated and control insects were then transferred to glass vials (10 insects per vial) and kept in the incubator. Insect mortality was checked after 24 h, and the LD_50_ values were calculated using Probit analysis by using SPSS 19.0 for Windows 7 [53].

The contact toxicity of the essential oil and pure compounds against *L. bostrychophila* was tested as described [7]. A serial dilution of the essential oil/compounds (five concentrations) was prepared in *n*-hexane. A 5.5-cm-diameter filter paper was treated with 300 µL of the solution of the essential oil and compounds. The treated filter paper after treated with solid glue was placed in a 5.5-cm-diameter Petri dish and 10 booklice were put on the filter paper by using a hair brush. A cover was put and all the Petri dishes were kept in the incubator. *n*-Hexane was used as a negative control. Five concentrations and five replicates of each concentration were used. Mortality of insects was observed after 24 h. The LD_50_ values were calculated by using SPSS 19.0 for Windows 7 [53].

### 3.7. Repellent Activity

The repellent effects of the essential oils against *T. castaneum* and *L. bostrychophila* were assessed by using assays on Petri dishes [54]. Petri dishes 9 cm in diameter were used to confine beetles during the experiment. The essential oil of *J. formosana* was prepared in *n*-hexane (78.63, 15.73, 3.15, 0.63, and 0.13 nL/cm^2^). Filter paper 9 cm in diameter was cut in half and 500 μL of each concentration was applied separately to half of the filter paper as uniformly as possible with a micropipette. The other half (control) was treated with 500 μL of absolute *n*-hexane. As for the booklice, the crude essential oil was diluted in *n*-hexane to different concentrations (63.17, 12.63, 2.53, 0.51, 0.10 nL/cm^2^). Petri dishes 5.5 cm in diameter were used to confine *L. bostrychophila*. Filter paper 5.5 cm in diameter was cut in half and 150 μL of each concentration was applied separately to half of the filter paper. The other half (control) was treated with 150 μL of *n*-hexane. For both tests, both the treated half and the control half were then air-dried to evaporate the solvent completely (30 s). Each reassembled filter paper after treatment with solid glue was pasted in a Petri dish. Twenty insects were released in the center of each filter paper disk, and a cover was placed over the Petri dish. Five replicates were used, and the experiment was repeated three times. Counts of the insects present on each strip were made after 2 and 4 h. The percent repellency (PR) of each volatile oil was then calculated using the formula:

PR (%) = [(Nc − Nt)/(Nc + Nt)] × 100
(1)

where Nc is the number of insects present in the negative control half and Nt is the number of insects present in the treated half. The averages were then assigned to different classes (0 to V) using the following scale (percentage repellency) [52]. Class, % repellency: 0, >0.01 to <0.1; I, 0.1–20.0; II, 20.1–40.0; III, 40.1–60.0; IV, 60.1–80.0; and V, 80.1–100. Analysis of variance (ANOVA) and Tukey’s test were conducted by using SPSS 19.0 for Windows 7. Percentage was subjected to an arcsine square-root transformation before ANOVA and Tukey’s test.

## 4. Conclusions

In this paper, we report contact and repellent activities of the three isolated constituents from the essential oil of *J. formosana* leaves against red flour beetles and booklice for the first time. These findings suggest that the essential oil of *J. formosana* leaves has potential to be developed into natural insecticides and repellents to control insects in stored products.

## Figures and Tables

**Table 1:** Chemical composition of the essential oil of *Juniperus formosana* leaves.

No.	RI ^a^	Compounds	Relative Area % ^b^	Identification Methods ^c^
1	931	α-Pinene	21.66	MS, RI, Co
2	973	β-Phellandrene	6.63	MS
3	996	Isoterpinolene	3.48	MS, RI
4	1018	*p*-Cymene	1.78	MS, RI
5	1030	Limonene	11.00	MS, RI, Co
6	1050	γ-Terpinene	3.49	MS, RI, Co
7	1073	2-Pentyl valerate	0.13	MS, RI
8	1099	5-Methyl-3-heptyne	0.28	MS, RI
9	1114	Thujone	1.34	MS, RI
10	1119	Campholenic aldehyde	0.71	MS, RI
11	1131	Pinocarveol	2.19	MS, RI
12	1136	2,5-Dihydrotoluene	0.30	MS
13	1138	trans-Pinocarveol	0.40	MS, RI
14	1158	2-Methyl-3-hexyne	0.22	MS, RI
15	1167	Borneol	0.18	MS, RI
16	1179	4-Terpineol	11.25	MS, RI, Co
17	1189	α-Terpineol	0.59	MS, RI
18	1196	Estragole	4.62	MS, RI, Co
19	1222	3-Ethyl-3-methyldecane	0.15	MS, RI
20	1234	Citronellyl formate	1.68	MS
21	1247	Carvone	0.18	MS, RI
22	1258	p-Menth-1-en-3-one	0.80	MS, RI
23	1268	Methyl citronellate	0.31	MS
24	1485	Germacrene D	0.18	MS, RI, Co
25	1528	δ-Cadinene	0.56	MS, RI
26	1550	Hedycaryol (and Elemol) ^d^	5.40	MS, RI
27	1608	Cedrol	3.59	MS, RI
28	1649	β-Eudesmol	0.83	MS, RI
		Total	83.93	

^a^ Retention index (RI) relative to the homologous series of (C_5_–C_36_) on the HP-5 MS capillary column; ^b^ Relative area (peak area relative to the total peak area); ^c^ MS = mass spectrum, Co = co-injection with standard compound; ^d^ Both hedycaryol and elemol (interconverted by Cope rearrangement) have the same MS and RI.

**Table 2:** Contact toxicity of *Juniperus formosana* essential oil and its constituents against *Tribolium* *castaneum* (TC) and *Liposcelis bostrychophila* (LB).

Insects	Treatments ^a^	LD_50_ (µg/Adult; µg/cm^2^)	95% FL (µg/Adult; µg/cm^2^)	Slope ± SE	Chi Square (χ^2^)	*p*-Value
TC	Essential oil	29.14	26.32–31.96	2.14 ± 0.23	18.40	0.736
	α-Pinene	25.37	20.67–29.13	1.28 ± 0.18	16.07	0.852
	4-Terpineol	7.65	6.75–8.55	2.21 ± 0.33	18.77	0.715
	d-Limonene	14.88	12.75–17.00	1.42 ± 0.17	19.83	0.652
	Pyrethrins ^b^	0.26	0.22–0.30	3.34 ± 0.32	13.11	0.950
LB	Essential oil	81.50	78.08–84.97	4.77 ± 0.50	14.70	0.905
	α-Pinene	873.73	830.73–921.29	8.27 ± 0.93	18.63	0.772
	4-Terpineol	29.50	28.19–30.87	4.23 ± 0.45	11.98	0.970
	d-Limonene	259.62	238.13–283.68	5.56 ± 0.57	16.10	0.851
	Pyrethrins ^c^	18.72	17.60–19.92	2.98 ± 0.40	10.56	0.987

^a^ The mortality of the control (*n*-hexane) was 0 µg/adult for TC and 0 µg/cm^2^ for LB; ^b^ Data from You *et al.* [36]; ^c^ Data from Yang *et al.* [37].

## Supplementary Materials

Supplementary materials can be accessed at: http://www.mdpi.com/1420-3049/21/4/504/s1. The NMR data for α-pinene (**1**), 4-terpineol (**2**) and d-limonene (**3**).
