# Supplementary material for: Contact and Repellent Activities of the Essential Oil from Juniperus formosana against Two Stored Product Insects
Source: Molecules. 2016 Apr 16;21(4):504. doi: 10.3390/molecules21040504 (PMC6273697; doi:10.3390/molecules21040504)
Supplement: Supplementary file 1 [file molecules-21-00504-s001.pdf]

Supplementary Materials: Contact and Repellent Activities of the Essential Oil from *Juniperus formosana* against Two Stored Product Insects

Shanshan Guo, Wenjuan Zhang, Junyu Liang, Chunxue You, Zhufeng Geng, Chengfang Wang and Shushan Du

The NMR data for α-pinene (**1**), 4-terpineol (**2**) and D-limonene (**3**).

α-Pinene (**1**). Colorless oil, $C_{10}H_{16}$. ^{1}H-NMR (500 MHz, $CDCl_3$) δ (ppm): 5.21 (1H, s, H-3), 2.36 (1H, m, H-7β), 2.21 (1H, dd, *J* = 20.0 Hz, H-4α), 2.17 (1H, dd, *J* = 20.0 Hz, H-4β), 2.09 (1H, s, H-5), 1.96 (1H, t, *J* = 5.0 Hz, H-1), 1.68 (3H, s, 10-CH_3), 1.29 (3H, s, 8-CH_3), 1.18 (1H, d, *J* = 5.0 Hz, H-7α), 0.86 (3H, s, 9-CH_3); ^{13}C-NMR (125 MHz, $CDCl_3$) δ (ppm): 144.6 (C-2), 116.0 (C-3), 47.0 (C-1), 40.7 (C-5), 38.0 (C-6), 31.5 (C-7), 31.3 (C-4), 26.4 (C-8), 23.0 (C-10), 20.8 (C-9).

4-Terpineol (**2**). Colorless oil, $C_{10}H_{18}O$. ^{1}H-NMR (500 MHz, $CDCl_3$) δ (ppm): 5.32 (1H, m, H-5), 2.17 (2H, m, H-6), 1.94 (2H, m, H-3), 1.71 (3H, s, 10-CH_3), 1.67 (1H, m, H-7), 1.58 (2H, m, H-2), 0.97 (3H, d, *J* = 7.0 Hz, 8-CH_3), 0.94 (3H, d, *J* = 7.0 Hz, 9-CH_3); ^{13}C-NMR (125 MHz, $CDCl_3$) δ (ppm): 133.88 (C-4), 118.45 (C-5), 71.75 (C-1), 36.78 (C-7), 34.64 (C-2), 30.78 (C-6), 27.07 (C-10), 23.27 (C-3), 16.83 (C-8, 9).

D-Limonene (**3**). Colorless oil, $C_{10}H_{16}$. ^{1}H-NMR (500 MHz, $CDCl_3$) δ (ppm): 5.43 (1H, m, H-6), 4.74 (2H, s, H-9), 2.05–2.16 (3H, m, H-2, H-4), 1.93–2.00 (2H, m, H-5), 1.81–1.85 (1H, m, H-3a), 1.76 (3H, s, H-10), 1.68 (3H, s, H-7), 1.46–1.54 (1H, m, H-3b); ^{13}C-NMR (125 MHz, $CDCl_3$) δ (ppm): 150.25 (C-8), 133.73 (C-1), 120.66 (C-6), 108.37 (C-9), 41.10 (C-4), 30.82 (C-2), 30.61 (C-5), 27.94 (C-3), 23.46 (C-7), 20.80 (C-10).
